# Influence of Monocalcium Phosphate on the Properties of Bioactive Magnesium Phosphate Bone Cement for Bone Regeneration

**DOI:** 10.3390/ma15062293

**Published:** 2022-03-20

**Authors:** Shaochun Lv, Tianyu Qu, Hisham Al-Ward, Liting Mu, Hongbin Qiu, Yunlong Zhang

**Affiliations:** 1 Key Laboratory of Microecology-Immune Regulatory Network and Related Diseases, School of Basic Medicine, Jiamusi University, Jiamusi 154007, China; acefire2008@163.com; 2 Department of Chemical Engineering & Material Science, Stevens Institute of Technology, Hoboken, NJ 07030, USA; tqu3@stevens.edu; 3 Shanghai Institute of Stem Cell Research and Clinical Translation, School of Medicine, Tongji University, Shanghai 200065, China; hisham_alward@yahoo.com; 4 School of Pharmacy, Jiamusi University, Jiamusi 154007, China; muliting@163.com; 5 School of Public Health, Jiamusi University, Jiamusi 154007, China; 6 School of Material Science & Engineering, Jiamusi University, Jiamusi 154007, China

**Keywords:** magnesium phosphate, bioactive bone cement, monocalcium phosphate, bone defects, orthopedic materials

## Abstract

Bone defects occurring for various reasons can lead to deformities and dysfunctions of the human body. Considering the need for clinical applications, it is essential for bone regeneration to exploit a scaffold with bioactive bone cement. In this study, we fabricated bioactive magnesium phosphate bone cement (BMPC) at room temperature; then, it was set at to °C and 100% humidity for 2 h. The process was as follows: Simulating a clinical environment, magnesium oxide (MgO) was formed by calcining basic magnesium carbonate (Mg_2_(OH)_2_CO_3_). MgO, potassium dihydrogen phosphate (KH_2_PO_4_) and carboxymethyl chitosan (C_20_H_37_N_3_O_14_, CMC) were mixed to form magnesium phosphate bone cement (MPC); then, monocalcium phosphate (Ca(H_2_PO_4_)_2_) was added to neutralize the alkaline product after MPC hydration to fabricate bioactive magnesium phosphate bone cement (BMPC). The influence of the doped content of Ca(H_2_PO_4_)_2_ on the properties of bone cement was discussed. The results showed that Ca(H_2_PO_4_)_2_ and CMC can adjust the setting time of bone cement to between 8 and 25 min. The compressive strength increased first and then decreased. After 48 h without additional pressure, the compressive strength reached the maximum value, which was about 38.6 MPa. Ca(H_2_PO_4_)_2_ and CMC can play a synergistic role in regulating the properties of BMPC. The BMPC was degradable in the simulated body fluid (SBF). The results of the cytotoxicity experiment and laser confocal microscopy experiment indicated that BMPC fabricated at room temperature had better biocompatibility and degradability, which was more consistent with clinical operation requirements. BMPC is a promising orthopedic material and is suitable for repairing bone defects.

## 1. Introduction

Treatments of bone tissue defects are a common problem in clinics. Currently, traditional autologous bone transplantation remains the “golden standard” clinical treatment of bone defects. However, autologous bone transplantation could increase trauma in patients. Sources of allogeneic bone from donors are limited, and there is a risk of immune rejection after bone grafting in vivo. In addition, the number of operations could also be increased, and the healing period may be prolonged. Future development prospects are not ideal [1,2]. Compared with traditional bone transplantation, degradable bioactive bone cement has been developed to repair bone defects, with the advantages of incurring minor damage due to bone tissue engineering technology. Proper repair of the bone morphology is necessary in the defect area, with no obvious antigenicity [3].

Monocalcium phosphate (Ca(H_2_PO_4_)_2_), also known as acidic calcium phosphate, is a colorless, granular, and crystalline powder. It exists in the form of Ca(H_2_PO_4_)_2_•H_2_O at room temperature. The aqueous solution is acidic and loses crystalline water after heating. It is widely used as a buffer and food additive [4].

Chitosan is the product of the deacetylation of chitin; it is the most important derivative of chitin. The surface of chitosan is hydrophilic. As a key water-soluble chitosan derivative, carboxymethyl chitosan (CMC) has good biocompatibility and biodegradability. It is widely used in hydrogels, wound healing biomaterials, and tissue engineering scaffold materials [5]. Previous studies have shown that CMC exhibits no antigenicity in animals and is used as an additive for bone defect repair materials and for the fabrication of bone cement [6].

MPC is an inorganic, non-metallic, bioceramic material generated through slightly soluble salinization reactions. It is commonly used in industrial applications. Based on its characteristics of high early strength and rapid curing, it has good biocompatibility and biodegradability. In recent years, it has attracted the attention of researchers exploring bioactive bone scaffolds [7]. MPC can be fabricated by mixing magnesium oxide (MgO) and ammonium dihydrogen phosphate (NH_4_H_2_PO_4_) as solid components. The main reaction product is magnesium ammonium phosphate (MgNH_4_PO_4_), commonly known as guano stone, a natural crystal [8]. However, this process leads to the release of ammonia (NH_3_) during the degradation of bone cement, which can easily contaminate the environment and is toxic to implanted tissue [9]. Potassium dihydrogen phosphate (KH_2_PO_4_) has been applied to replace NH_4_H_2_PO_4_ in the fabrication of industrial cement [10]. Compared with NH_4_H_2_PO_4_, KH_2_PO_4_ has a smaller dissociation constant and lower solubility; thus, it is easier to control the reaction rate. Additionally, it does not produce NH_3_ when reacting with water. The final product is magnesium potassium phosphate (MgKPO_4_), which is isomorphic with guanite [11]. However, in the field of bioactive bone cement studies, there have been few reports on the determination of the pH of MgKPO_4_ in the aqueous phase or the effect of Ca(H_2_PO_4_)_2_ on the properties of BMPC by simulating the clinical environment, i.e., keeping the room sterile and the temperature at 25 °C. Furthermore, although some references have mentioned that Ca(H_2_PO_4_)_2_ can reduce the hydration reaction rate to a certain extent, we cannot yet acquire satisfactory properties of bone cement through repeat tests; therefore, the improvements in properties are still very limited by only adding Ca(H_2_PO_4_)_2_ to MPC. For example, the compressive strength of MPC through repeat tests cannot achieve the mean value (13.6MPa) of the strength of human cancellous bone, meaning that it is difficult to meet the clinical needs of bone grafts in vivo. Therefore, in addition to Ca(H_2_PO_4_)_2_, it is necessary to combine it with other bioactive components (e.g., carboxymethyl chitosan) to improve the properties of MPC.

Temperature conditions should not be neglected during the fabrication of bone cement. Recently, several studies have preferred the preparation of inorganic salt-based bone cement at low temperatures such as below 0 °C [12]. However, the low-temperature conditions do not match the room temperature conditions required for surgery. Moreover, the cost of creating a low-temperature condition is relatively high. Additionally, the properties of such bone cement are still uncertain due to a lack of sufficient evidence, and its application prospect is still limited. Given that the main components of industrial cement are inorganic salts, and that the main components of MPC are similar to those of industrial cement, together with the principles of preparation originally derived from industrial cement. Therefore, we reviewed the literature on the effect of low temperature on the properties of industrial cement [13,14]. It has been reported that the properties of magnesium phosphate cement fabricated at low temperatures in civil engineering are not optimal. Firstly, crystals can be produced inside the cement, which will damage the cement, and cracks are easy to appear on its surface. Secondly, the compressive strength of cement can be restricted by low temperature. The lower the temperature, the slower the growth in the early compressive strength with cement. Thirdly, the hydration rate of cement is slowed down; therefore, it does not easily solidify. Therefore, considering the possibility of bone cement applications in the human body, we need to maintain a cautious attitude towards bone cement fabricated at low temperatures. In a hospital, the temperature in operating rooms is generally controlled at 20~26 °C [15,16]. Thus, it is necessary that the solid and liquid phases of bone cement are mixed and stirred at room temperature and quickly implanted into the bone defect. Therefore, we should pay more attention to improving the properties of bone cement materials at room temperature, in order to avoid the potential risks.

In this study, BMPC was investigated by simulating a clinical environment. Firstly, MgO, KH_2_PO_4_ and CMC were mixed at 25 °C in a sterile room to fabricate bioactive magnesium phosphate bone cement (BMPC), and the pH of the MPC hydration product MgKPO_4_ was measured. Then, Ca(H_2_PO_4_)_2_ was added to MPC. The acidity produced by the degradation of Ca(H_2_PO_4_)_2_ was utilized to neutralize the alkalinity of MgKPO_4_, the main hydration product of MPC. Finally, the effects of Ca(H_2_PO_4_)_2_ and CMC on the pH, preserving time, compressive strength, degradability, cell morphology, and biocompatibility of bone cement are discussed.

## 2. Materials and Methods

### 2.1. Fabrication of BMPC Samples

BMPC is composed of solid powder and liquid phase (deionized water). The solid powder was made from MgO, KH_2_PO_4_, CMC and Ca(H_2_PO_4_)_2_. Among them, MgO is prepared through the heating and decomposition of basic magnesium carbonate (Mg_2_(OH)_2_CO_3_). All powder materials were obtained from Sinopharm Chemical Reagent Co., China. Mg_2_(OH)_2_CO_3_ was calcined in a muffle furnace at 1500 °C with a heating rate of 10 °C/min; then, it was kept warm for 2 h, cooled in the furnace, wet-ball-milled with alcohol for 2 h, then sieved through 300-mesh nylon sieve to prepare MgO. KH_2_PO_4_ and Ca(H_2_PO_4_)_2_ were screened through a 300-mesh nylon screen after ball milling. Then, CMC powder was added by 1.5% of mass fraction [17]. Before the solid and liquid phases were mixed, the room was irradiated with an ultraviolet lamp for 60 min. At 25 °C, deionized water was added at a solid:liquid ratio of 1.6 g/mL, after being thoroughly mixed, made into a bioactive cement paste, and placed in a 3D printing polyethylene mold (size 10 × 10 × 5 mm) without applying additional pressure. The BMPC sample was collected after restoration at 37 °C and 100% relative humidity for 48 h. The composition of BMPC samples was analyzed by X-ray crystal diffraction (XRD, D8-Advance, Bruker Co., Karlsruhe, Germany). The surface morphology and microstructure of BMPC were observed and studied with scanning electron microscopy (SEM, JSM-7800, JEOL Ltd., Tokyo, Japan).

### 2.2. pH Determination of MPC Hydration Product

Four different mass ratios of MgO and KH_2_PO_4_, i.e., 1:2, 1:3, 1:4 and 1:5, were selected. CMC was added by a 1.5% mass fraction and synthesized MPC with the participation of deionized water. The main hydration product MgKPO_4_ was analyzed by XRD. The MPC sample was immersed in normal saline with a solid:liquid ratio of 0.2 g/mL and placed in a constant temperature oscillator for 24 h. The supernatant was removed, and the pH was measured. Ca(H_2_PO_4_)_2_ was introduced at a mass ratio of 1:2 (MgO and KH_2_PO_4_) in order to prepare different mass fractions of Ca(H_2_PO_4_)_2_ bone cement samples (0 wt.%, 20 wt.%, 40 wt.%, and 60 wt.%), which were recorded as BMPC0, BMPC20, BMPC40 and BMPC60, respectively. The effects of different contents of Ca(H_2_PO_4_)_2_ on the properties of BMPC were examined.

### 2.3. Characterization of BMPC Samples

In order to determine the pH of the BMPC soaking solution with different contents of Ca(H_2_PO_4_)_2_, BMPC samples were immersed in normal saline at a solid:liquid ratio of 0.2 g/mL and stored in a constant temperature oscillator for 24 h. The supernatant was taken, and the pH was determined with a pH meter. The setting time of BMPC was measured using a Vicat meter. The Vicat meter had a round metal rod with a weight of 300 g and a test needle 1mm in diameter and 50 mm in length at the lower end of the rod. The setting time was the time required from mixing the solid and liquid phases of the composite bone cement to when the test needle failed to penetrate more than 1 mm into the specimen. The test was repeated twice, and the average value was calculated. After setting the bone cement for 48 h, the compressive strength was measured with a loading rate of 2 mm/min using the MTS810 universal mechanical testing machine, and five samples were taken for each group. The degradation of different BMPC in simulated body fluid (SBF) was determined from the degradation rate at different time points. BMPC samples (10 mm × 10 mm × 5 mm) were dried for 2 h, and the initial weight was recorded as W_0_. Then, BMPC samples were immersed in SBF at 37 °C in a thermostatic shaker with a solid:liquid mass ratio of 1:20 g/mL. The solution was renewed every two days. The weights of BMPC were determined on days 3, 5, 7, 14, 21 and 28. The operation was performed by removing the specimen from the liquid after immersion, rinsing it with deionized water, drying it for 2 h, and recording the new weight of all specimens. All values were the average of three tests. The degradation rate was calculated by using the following Formula (1):

Degradation rate = (W_0_ − W_t_)/W_0_ × 100% (1)

### 2.4. Cell Culture and Cytotoxicity Experiments

Mouse 3T3E1 osteoblasts were selected and cultured in complete Roswell Park Memorial Institute 1640 (RPMI) medium containing 10% fetal bovine serum (FBS) and 1% antibiotics (penicillin and streptomycin) at 37 °C in a humidified incubator with 5% CO_2_. Cells were harvested after the confluence with 0.25% trypsin and inoculated individually on culture dishes at an initial density of 2000 cells per well, placed in 96-well culture dishes, and incubated at 37 °C/CO_2_. The culture medium was changed every 3 days. BMPC extracts with different mass fractions of Ca(H_2_PO_4_)_2_ were used as the experimental group, and a normal cell culture medium was used as the control group. The BMPC extracts were prepared according to the method presented in the literature [18]. First, the bone cement raw material was added to serum-free RPMI at a solid:liquid ratio of 0.2 g/mL under aseptic conditions to obtain the solution, which was incubated at 37 °C for 24 h and then centrifuged; the supernatant was collected, refrigerated at 4 °C, and stored for further use. The cells were cultured for 1 day, 3 days, and 5 days. The cytotoxicity of BMPC was estimated with the MTT (3-(4,5-dimethylthiazole-2-yl)-2,5-diphenyl-2H -tetrazolium bromide) assay. To each well, 20 μL of MTT solution was added, and incubation was continued at 37 °C in 5% CO_2_ for 4 h. The supernatant in the wells was then discarded, and 200μL of dimethyl sulfoxide (DMSO) was added to fully dissolve the purple crystals. On days 1, 3, and 5, the absorbance value of each well at 490 nm was measured by an enzyme maker: OD. The relative growth rate (RGR) was calculated according to the measured OD value with Equation (2), and the test results were evaluated according to the cytotoxicity grade standard (Table 1) [19].

RGR(%) = A_test_/A_control_ × 100% (2)

### 2.5. Laser Confocal Microscopy Experiment

Mouse 3T3E1 osteoblasts in the logarithmic growth stage were taken at 37 °C, 5% CO_2_ in a constant temperature oven, and treated with 4 × 10^5^/mL in four groups BMPC extracts with different levels of Ca(H_2_PO_4_)_2_, respectively. After 5 days of culture, the cells were fixed with 0.2 mL of 4% paraformaldehyde, and the wells were placed in the thermostat for 10 min. The fixative solution was pipetted out, rinsed with phosphate-buffered saline (PBS), and 0.5% Triton x-1000 5 mL was added. The membrane was permeabilized in the thermostat for 20 min, after which the permeabilized solution was pipetted out, rinsed with PBS, and 0.5 mL of staining solution was added to re-stain the plates for 5 min; then, the re-staining solution was pipetted out and rinsed with PBS. Subsequently, 200 μL of rhodamine ghostpen ring peptide was added and treated in a dark room at room temperature for 40 min, then sealed with fluorescence quencher after PBS rinsing. After material treatment, cell morphology and growth were observed using laser confocal microscopy.

## 3. Results

### 3.1. Hydration Products and pH of MPC

**Figure 1:**
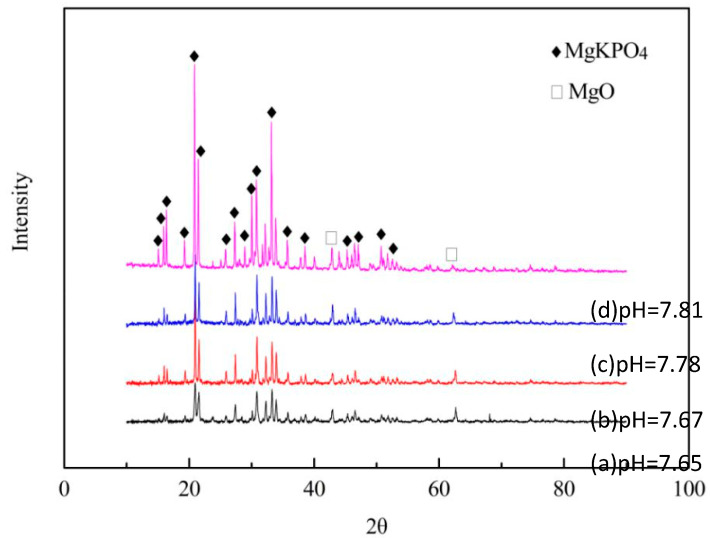
The hydration products and pH of MPC specimens. (a) 1:2; (b) 1:3; (c) 1:4; (d) 1:5.

Figure 1 shows the XRD and pH results of MgO and KH_2_PO_4_ (MPC) at different mass ratios. According to the analysis of the XRD data, all the hydration products of MPC specimens were composed of MgO and KMgPO_4_ (Figure 1), and the main diffraction peaks were KMgPO_4_. However, the peak of CMC was not revealed. The diffraction peaks of MgO were weak, which indicated that there was only a small amount of MgO. The diffraction peak intensity of MgO decreased with the increase in KH_2_PO_4_, as the diffraction peak intensity of KMgPO_4_ enhanced with the increase in KH_2_PO_4_. Therefore, it indicated that MgO was almost completely transformed into KMgPO_4_. With the increase in KMgPO_4_, the pH of the MPC soaking solution was enhanced, indicating that KMgPO_4_ was alkaline.

### 3.2. The SEM Images of BMPC Specimens

**Figure 2:**
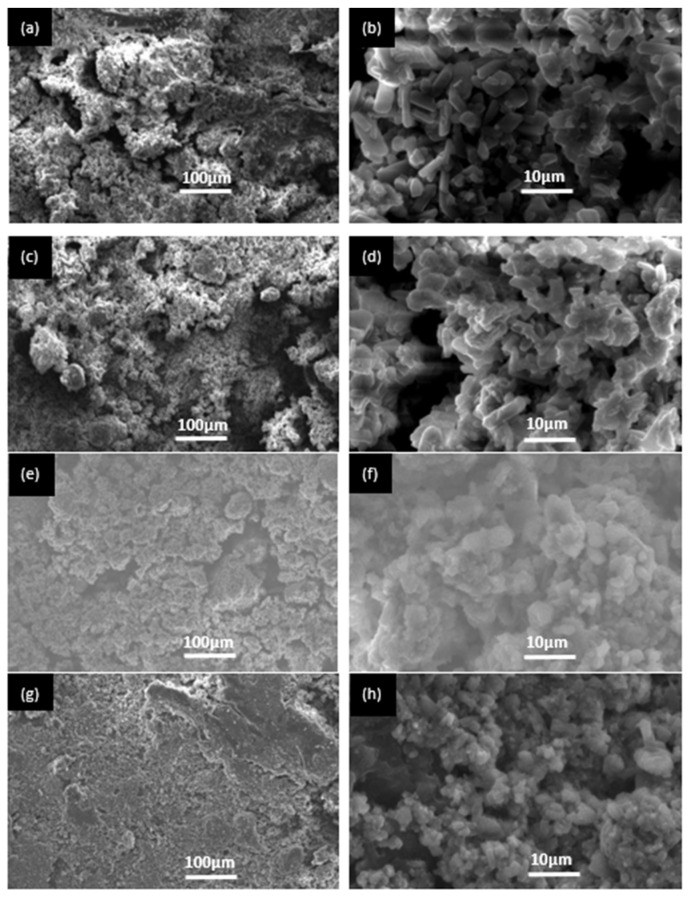
SEM images of BMPC samples. (**a**,**b**) BMPC0; (**c**,**d**) BMPC20; (**e**,**f**) BMPC40; (**g**,**h**) BMPC60.

Figure 2 shows the surface morphology of BMPC specimens with different contents of Ca(H_2_PO_4_)_2_ after setting for 48 h. It was found that BMPC0 contained prismatic crystals. It was speculated that these crystals were KMgPO_4_, as well as clay materials with dense morphology and structure, as shown in Figure 2a,b. With the increase in Ca(H_2_PO_4_)_2_ content, prismatic crystals disappeared, and clay materials increased, which was consistent with the XRD results. KMgPO_4_ disappeared with the increase in Ca(H_2_PO_4_)_2_. It could be seen that clay materials accumulated together, resulting in high strength of the specimens, as shown in Figure 2c–f. Moreover, there were many pores in the clay materials, which was consistent with the experimental results observed by Wang et al. (2019) [20]. The existence of these pores was not only conducive to the degradation of bone cement and phosphate deposition, but also conducive to the adhesion and proliferation of osteoblasts; thus, the growth of new bone was induced. Therefore, it could result in the degradation of BMPC specimens Figure 2g,h.

### 3.3. The Characterization Results of BMPC Samples

**Figure 3:**
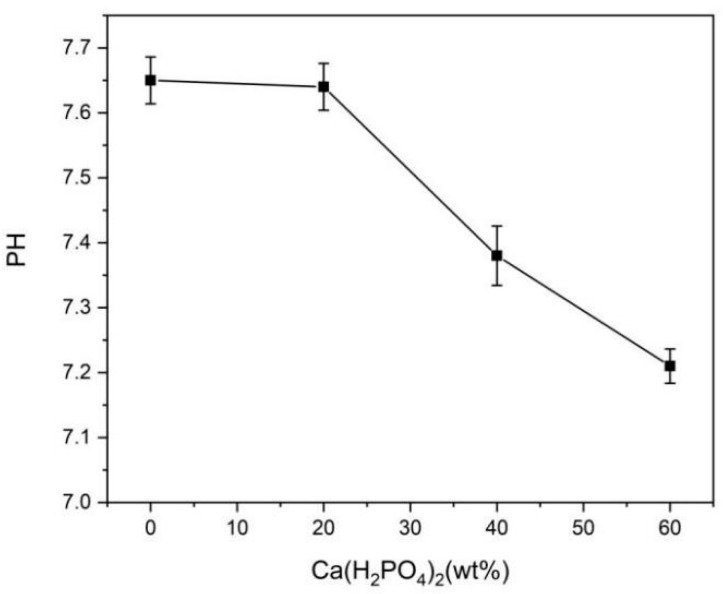
pH of BMPC extracts with Ca(H_2_PO_4_)_2_.

The pH values of BMPC extracts are shown in Figure 3. The pH decreased with the increases in Ca(H_2_PO_4_)_2_ contents, which was related to the hydration product after 48 h. With the increase in Ca(H_2_PO_4_)_2_ content, the KMgPO_4_ phase and MgO phase disappeared as the MgHPO_4_ phase appeared, which resulted in a decrease in the pH of the BMPC extracts. This showed that KMgPO_4_ was alkaline during immersion. Moreover, the research results showed that the pH of BMPC extracts with 40% or more Ca(H_2_PO_4_)_2_ decreased to less than 7.40, which was close to the pH value of SBF used in general cell experiments. These environmental pH conditions promoted cell growth and proliferation.

**Figure 4:**
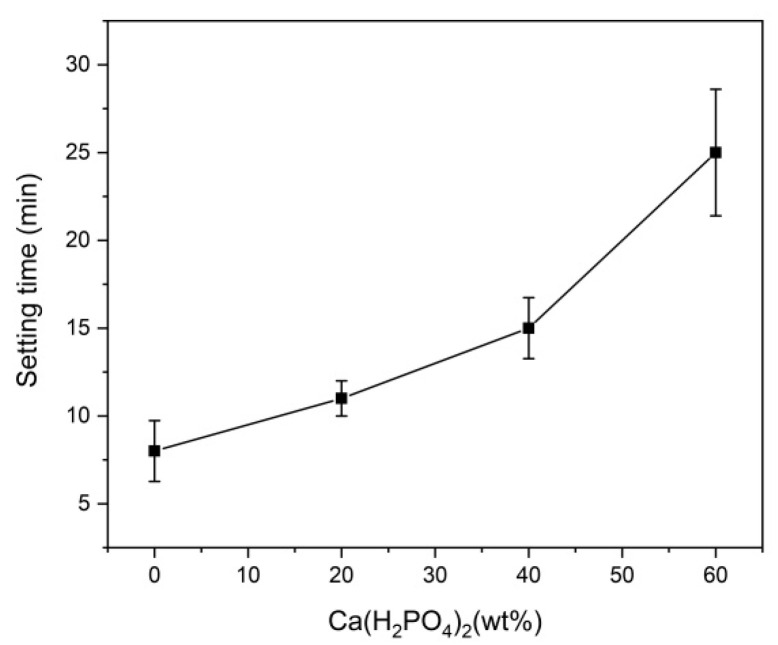
The setting time of BMPC specimens with Ca(H_2_PO_4_)_2_.

The effects of different amounts of Ca(H_2_PO_4_)_2_ on the setting time are shown in Figure 4. With the increasing Ca(H_2_PO_4_)_2_ contents, the setting time of BMPC increased from 8 min to 25 min, a time range which was consistent with the operating time needed for general clinical bone defect repairs. When the ratio of MgO to KH_2_PO_4_ was 1:2, the content of MgO was relatively overreacted (Table 2). In contrast, as the Ca(H_2_PO_4_)_2_ content increased, the acidity of the reaction system increased, more crystalline products were generated, and the main product, KMgPO_4_, disappeared, prolonging the time taken for the reaction to reach equilibrium and resulting in a longer setting time of the bone cement, facilitating its implantation.

**Figure 5:**
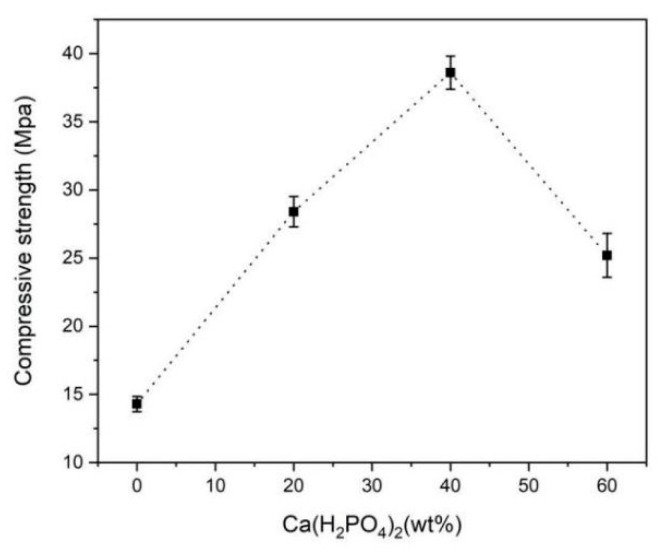
The compressive strength of BMPC specimens with Ca(H_2_PO_4_)_2_.

Figure 5 shows the effect of Ca(H_2_PO_4_)_2_ content on the compressive strength of BMPC specimens. After BMPC slurry was prepared at 25 °C and statically set for 48 h, the compressive strength initially increased, then decreased with the increase in Ca(H_2_PO_4_)_2_ content without any additional pressure being exerted. The compressive strength of BMPC40 reached the maximum value of 38.6 MPa, which was 31.4 MPa higher than that of magnesium ammonium phosphate bone cement prepared at low temperature [20]. The increase in compressive strength was due to the decrease in pH in the reaction system, resulting in more hydration products similar to clay particles (Figure 2). These granular materials were closely staggered and stacked together to yield high compressive strength. Therefore, the specimens not only exhibited satisfactory mechanical strength, but also met the needs for the on-site fabrication of bone cement at room temperature. Compared with BMPC40, the compressive strength of BMPC60 decreased. This was the reason that the crystal structure became irregular as the crystallinity of hydration products decreased (Figure 2), which did not yield a high compressive strength; therefore, the compressive strength was reduced with BMPC60.

After the bone cement was set for 48 h, KMgPO_4_ and surplus unreacted MgO were examined in the reaction system of BMPC0. With the increase in Ca(H_2_PO_4_)_2_ content, MgO gradually disappeared, and MgHPO_4_, Mg_3_(PO_4_)_2_, Ca_3_(PO_4_)_2_, and Ca_10_(PO_4_)_6_(OH)_2_ appeared. KMgPO_4_ disappeared in the reaction system with more than 40%Ca(H_2_PO_4_)_2_. Finally, Mg_3_(PO_4_)_2_, MgHPO_4_, Ca_10_(PO_4_)_6_(OH)_2_, and MgKH(PO_4_)_2_ were formed. Ca_10_(PO_4_)_6_(OH)_2_ is also known as hydroxyapatite (HAP), an inorganic component of human bone.

After degrading for 28 days, Mg_3_(PO_4_)_2_ could be examined in all specimens. Among them, the degradation products of BMPC with Ca(H_2_PO_4_)_2_ included Mg_3_(PO_4_)_2_, MgHPO_4_, pyrophosphate and HAP. Notably, BMPC filled with Ca(H_2_PO_4_)_2_ produced MgHPO_4_ after setting for 48 h. After degradation for 28 days, MgHPO_4_ remained, whereas other products were transformed into magnesium phosphate, pyrophosphate, and HAP (Table 2). Thus, MgHPO_4_ could be regarded as a buffering agent in the reaction system, and the regulation of pH with BMPC depended on the existence of MgHPO_4_.

**Figure 6:**
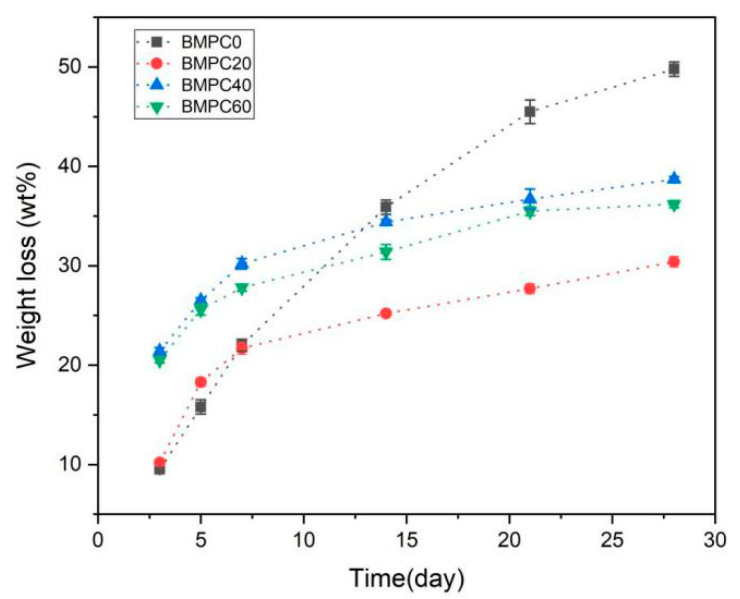
The degradation rate of BMPC samples with Ca(H_2_PO_4_)_2_.

Figure 6 shows the degradation rate of BMPC samples soaked in SBF at different times. Demonstrably, BMPC degraded in SBF as time passed, and the degradation rate was related to the amount of product (Table 2). After 28 days of degradation, the amount of product from BMPC40 samples exhibited the most significant reduction and the highest degradation rate. The results showed that the more products disappeared, the faster the degradation.

### 3.4. The Cytotoxicity Results with BMPC by MTT Assay

**Figure 7:**
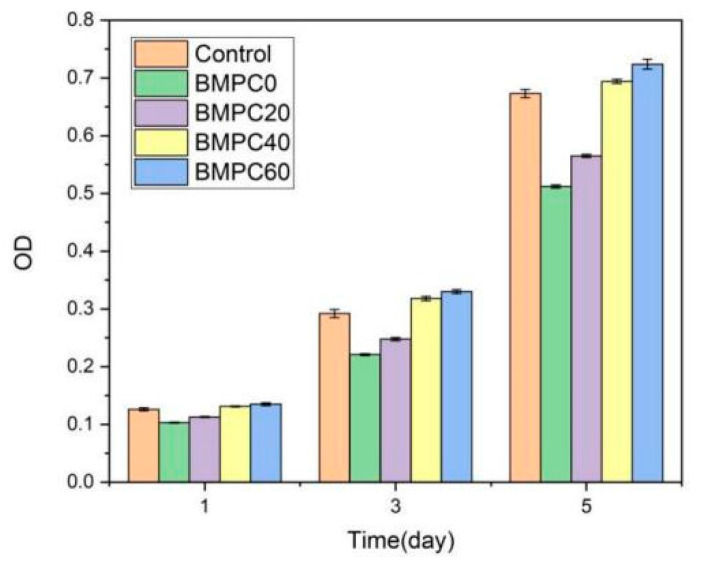
The cytotoxicity of BMPC samples.

MTT assays are widely applied to detect the bioactivity or cytotoxicity of biomaterials. An MTT assay was selected to detect the extracts of BMPC samples to determine the cytotoxicity to mouse 3T3E1 osteoblasts. According to RGR and toxicity grade standards (Table 3), when the Ca(H_2_PO_4_)_2_ content was less than 40%, the toxicity levels of the BMPC samples was categorized as grade 1. When the Ca(H_2_PO_4_)_2_ content reached 40% or more, the toxicity grade of the BMPC samples was grade 0 (Figure 7, Table 3). This indicated that BMPC40 and BMPC60 samples had good biocompatibility, which was consistent with the pH results of the BMPC extracts (Figure 3). Moreover, the addition of Ca(H_2_PO_4_)_2_ reduced the pH values of BMPC extracts, which were closer to the pH of simulated body fluid; therefore, it is beneficial to improve the biocompatibility of bone cement. It also proved that the alkaline environment of KMgPO_4_ was not suitable for the growth, adhesion, or proliferation of mouse 3T3E1 osteoblasts.

### 3.5. The Laser Confocal Microscopy Results

**Figure 8:**
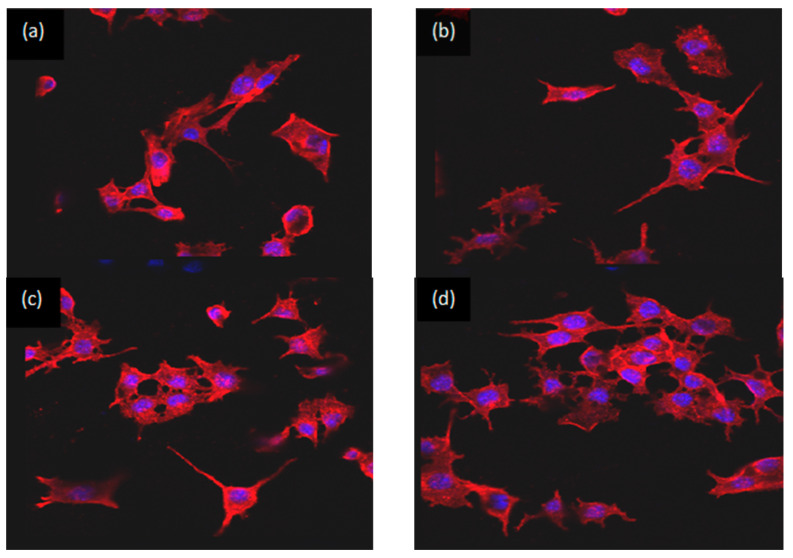
Images of the laser confocal microscopy experiment of BMPC samples. (**a**) BMPC0; (**b**) BMPC2; (**c**) BMPC40; (**d**) BMPC60.

The experimental laser confocal microscopy results are shown in Figure 8. The morphology of mouse 3T3E1 osteoblasts in bone cement extracts was irregular, mostly triangular and polygonal, with many protrusions, and were mononuclear with an oval nucleus. The cell matrix was wrapped around the nucleus, and the pseudopodia between cells fused with each other. This indicated that the cells had grown on the matrices of all bone cement samples. In terms of different Ca(H_2_PO_4_)_2_ contents, compared with BMPC0 Figure 8a and BMPC20 Figure 8b, the numbers of osteoblasts with BMPC40 and BMPC60 increased significantly Figure 8c,d. This suggested that with the increase in Ca(H_2_PO_4_)_2_ content, changes in the bone cement degradation products and the alkaline environment promoted cell proliferation and differentiation. This was consistent with the cytotoxicity results of BMPC from the MTT assay. Compared with the other groups, most cells were detected in BMPC60 extracts Figure 8c,d, which could be interpreted as occurring for two reasons. On the one hand, the pH values of BMPC40 and BMPC60 were close to the pH of simulated body fluid (Figure 3), which was suitable for cell growth and proliferation. On the other hand, the contents of calcium ions, magnesium ions, and phosphate ions in the solution were high, which provided a suitable environment for the growth of osteoblasts.

## 4. Discussion

Here, we developed novel BMPC with improved physicochemical properties by combining different ratios of Ca(H_2_PO_4_)_2_ and a fixed ratio of CMC with MPC. Through X-ray diffraction (XRD), it was revealed that the mass ratio of magnesium oxide (MgO) to potassium dihydrogen phosphate (KH_2_PO_4_) was 1:2. In the hydration process, there was a rapid reaction between MgO and KH_2_PO_4_. When the concentrations of potassium ions (K^+^), magnesium ions (Mg^2+^), and phosphate ions (PO_4_^3−^) in the solution reached a certain value, many hydration products, including MgKPO_4_, began to form through the interaction of ionic bonds. The ion reaction equation of the hydration reaction is:

K^+^ + Mg^2+^ + PO_4_^3−^ = MgKPO_4_ (3)

In addition to the MgKPO_4_ and unreacted MgO, XRD analysis did not reveal any other hydration products. It indicated that CMC mainly performed as a micro-filler in the MPC reaction system; therefore, it was not involved in the formation of hydration products. After adding different concentrations of Ca(H_2_PO_4_)_2_, the typical peaks of MgO and KMgPO_4_ gradually disappeared in the XRD analysis, indicating that all samples had been transformed into other hydration products.

The setting time was a vital property which was reflective of the polymerization time for repairing bone defects [21]. The setting time of MPC was greatly affected by conditions including the powder size, surface area, MgO content, powder-to-liquid ratio, etc. [22]. In the preparation process of inorganic salt bone cement, the acid-base reaction rate was rapid and difficult to control; thus, the setting time was very short [23]. Several prior studies have revealed that a setting time between 8 and 20 min is suitable for the surgical implantation of bone cement [23]. This study showed that the setting time could be prolonged from 8 min to 25 min by adjusting the content of Ca(H_2_PO_4_)_2_ in MPC, with CMC at a fixed ratio. Therefore, it was sufficiently long for repairing bone defects by injecting and shaping the cement. The setting time was prolonged because Ca(H_2_PO_4_)_2_ an acid salt; it exerted a buffering effect in the reaction system, which decelerated the rate of the hydration reaction. Moreover, CMC can form coatings to cover the surface of MgO and KH_2_PO_4_, slowing down the hydration reaction rate. We speculated that Ca(H_2_PO_4_)_2_ can coordinate with CMC in this process. Without a buffer, the hydration reaction can produce violent exothermic effects [24]. The prolonged setting time indicated that the composite cement exhibited a moderate hydration reaction, generating less heat during the setting process. This could be beneficial, avoiding tissue damage and apoptosis [25].

Bone cement needs to achieve a certain mechanical strength in clinical applications that can at least be comparable with the compressive strength of cancellous bone [26]. We found that the compressive strength first increased and later decreased with the increasing Ca(H_2_PO_4_)_2_ contents. The compressive strength of BMPC40 was nearly three times greater than that of human cancellous bone; therefore, it is suitable for use in repairing non-load bearing bone defects. The increase in compressive strength was due to the decrease in pH value in the reaction system, resulting in more hydration products similar to clay particles. These granular materials were closely staggered and stacked together to yield high compressive strength. Therefore, BMPC not only exhibited good mechanical strength, but also met the needs for the on-site preparation of bone cement at room temperature. Compared with BMPC40, the compressive strength of BMPC60 decreased. This was because the crystallinity of the hydration product of BMPC60 reduced, the crystal structure became irregular, resulting in a decrease in compressive strength. In addition, CMC, as a hydrophilic polymer, may adsorb deionized water in the liquid phase, forming a high-viscosity coating on the surface of cement. This can be observed from the SEM images. MPC exhibited many brittle crystal cracks, exhibit CMC filled these cracks, forming a dense microstructure, which had a certain degree of fracture resistance.

pH should also be considered, because it can significantly affect the osteogenesis of bone cement [27]. Generally, surplus MgO in MPC composites can lead to a large amount of OH^-^ being produced. In order to decrease the alkalinity of the hydration products, a relatively low Mg:P ratio of 1:2 was adopted. We also utilized the acidic characteristics of Ca(H_2_PO_4_)_2_ in aqueous solution to decrease the pH. With the increase in Ca(H_2_PO_4_)_2_ content, the KMgPO_4_ phase and MgO phase disappeared; as MgHPO_4_ phase appeared, the pH of the BMPC extracts decreased. Moreover, results showed that the pH of BMPC extracts with Ca(H_2_PO_4_)_2_ contents of 40% or more decreased to less than 7.40, which was close to the pH value of SBF used in general cell experiments. In addition, CMC is an amphoteric ether derivative with active groups such as hydroxyl(-OH), carboxyl(-COOH), and amino(-NH_2_); it can also decrease the pH of BMPC. We speculated that Ca(H_2_PO_4_)_2_ and CMC play a synergistic role in pH regulation.

The degradation rate and biodegradability are additional important properties of bone cement. There is evidence that a lower degradation rate may be caused by a lower porosity [28]. We found that the degradation rate was faster after Ca(H_2_PO_4_)_2_ was initially added to MPC at one week. Then, the degradation rates of Ca(H_2_PO_4_)_2_ groups gradually reduced, although the increasing trend was still present. We speculated that the Ca(H_2_PO_4_)_2_ acted as a buffer in the degradation process. Therefore, this reaction process can be controlled in a comparatively moderate condition. CMC may also play a synergistic role; however, this still needs to be tested in future studies. We observed that, when the Ca(H_2_PO_4_)_2_ content was 60%, the degradation rate decreased slightly compared with BMPC40. Differences in the degradation and pH of BMPC60 may be caused by the direct dissolution of Ca(H_2_PO_4_)_2_ after binding saturation. Studies have shown that Mg^2+^ ions have properties similar to those of bone tissue and display antibacterial activity, excellent biocompatibility, and biodegradability [29,30]. Water can penetrate the BMPC scaffold, allowing Mg^2+^ to diffuse. The release of Mg^2+^ was generally positively correlated with the degradation of BMPC, and the precipitation trend in Mg^2+^ achieved a stable state. Therefore, in this study, under the conditions of a controllable degradation rate, Mg^2+^ ions may be coordinated with calcium ions (Ca^2+^), acting as the nuclei for forming hydroxyapatite to achieve bone matrix mineralization.

In this study, in vitro cytotoxicity experiments and laser confocal microscopy experiments were used to evaluate the cellular response to biomaterials, identifying the cell morphology and internal structure. The results of the in vitro cell culture showed that BMPC composites exhibited no cytotoxicity. Mouse 3T3E1 cells proliferated and adhered to BMPC composites better than MPC (BMPC0). In addition, compared with MPC, the cell viability on BMPC increased with the additional Ca(H_2_PO_4_)_2_ content. The osteogenic response to bone cement is very important for osteanagenesis. Our results indicated that BMPC has good biocompatibility and osteogenesis characteristics. The BMPC introduced here presents suitable features for its use as a bioactive material for treating bone defects and inducing bone regeneration. However, the toxicity and other biological features of BMPC in vivo remain uncertain and should be explored in further investigations.

## 5. Conclusions

Here, we developed a degradable self-setting BMPC by combining Ca(H_2_PO_4_)_2_ with CMC and simulating a clinical environment. With the increase in Ca(H_2_PO_4_)_2_ content, the setting time of BMPC was prolonged from 8 min to 25 min, which was in line with the requirements for bone cement implantation. The compressive strength initially increased, and then decreased. After setting for 48 h, the maximum compressive strength reached 38.6 MPa, which was comparable with the compressive strength of non-load-bearing human bone. BMPC could be degraded in the simulated body fluid. After degradation, it produced magnesium phosphate and HAP, which was conducive to the formation of autologous bone. Cytotoxicity and laser confocal microscopy analyses of the BMPC extracts showed that the BMPC samples exhibited good biocompatibility. Additionally, the preparation cost of the BMPC is less than that of the current preparation technology at low temperature; thus, it is suitable for wide use. The results demonstrated that the BMPC fabricated in this study is a suitable bioactive material with potential value for clinical applications. However, because in vivo toxicology and osteogenesis were not investigated, nor were biological interactions at gene level assessed, the results of the current study do not represent complete evidence; hence, additional investigations are required.

## Figures and Tables

**Table 1:** Relative growth rate (RGR) and cytotoxicity grade standard.

RGR (%)	Toxicity Grade
≥100	0 Grade
75~99	1 Grade
50~74	2 Grade
25~49	3 Grade
1~24	4 Grade
0	5 Grade

**Table 2:** The hydration products of BMPC after setting for 48 h and soaking for 28 d tested by XRD.

	BMPC0	BMPC20	BMPC40	BMPC60
Setting for 48 h	①②	①②③④⑤⑥	③④⑤⑥⑦	③⑤⑥⑦
Soaking for 28 d	➊➍➎	➊➋➌➏➐➑	➊➌➏➑	➊➌➏➑

Setting for 48 h: ① MgO; ② MgKPO_4_; ③ Mg_3_(PO_4_)_2_; ④ Ca_3_(PO_4_)_2_; ⑤ MgHPO_4_; ⑥ Ca_10_(PO_4_)_6_(OH)_2_; ⑦ MgKH(PO_4_)_2_. Soaking for 28 d: ➊ Mg_3_(PO4)_2_; ➋ KMgPO_4_; ➌ MgHPO_4_; ➍ Mg_2_P_2_O_7_; ➎ K_4_P_2_O_7_; ➏ K_2_CaP_2_O_7_; ➐ Ca_3_(PO_4_)_2_; ➑ Ca_10_(PO_4_)_6_(OH)_2_.

**Table 3:** The relative growth rate (RGB) and toxicity grade (TG) of BMPC samples.

Group	1 d		3 d		5 d	
	RGB (%)	TG	RGB (%)	TG	RGB (%)	TG
BMPC0	81.75	1	75.68	1	76.08	1
BMPC20	89.68	1	84.93	1	83.95	1
BMPC40	103.97	0	108.90	0	103.12	0
BMPC60	107.14	0	113.01	0	107.58	0

## Data Availability

The data that support the findings of this study are available within the manuscript. Additional information can be provided from the corresponding author upon reasonable request.
